# Multisource spectral fusion combined with variable selection for rapid geographical origin discrimination of *Salvia miltiorrhiza*

**DOI:** 10.3389/fchem.2025.1730996

**Published:** 2025-12-09

**Authors:** Yue Jiao, Xiaoming Wu, Qi Wang, Xinjing Gui, Jing Yao, Xiaoying Duan, Ruixin Liu

**Affiliations:** 1 Department of Pharmacy, The First Affiliated Hospital of Henan University of Chinese Medicine, Zhengzhou, China; 2 School of Pharmacy, Henan University of Chinese Medicine, Zhengzhou, China; 3 Henan Province Engineering Research Center for Clinical Application, Evaluation and Transformation of Traditional Chinese Medicine, Zhengzhou, China; 4 Henan Provincial Key Laboratory for Clinical Pharmacy of Traditional Chinese Medicine, Zhengzhou, China; 5 Henan Institute for Drug and Medical Device Inspection, Zhengzhou, China; 6 NMPA Key Laboratory for Quality Control of Traditional Chinese Medicine (Chinese Materia Medica and Prepared Slices), Zhengzhou, China; 7 Provincial and Ministerial Co-construction Collaborative Innovation Center for Prevention and Treatment of Respiratory Diseases with Traditional Chinese Medicine of Henan University of Chinese Medicine, Zhengzhou, China

**Keywords:** mid-infrared spectroscopy(MIR), multisource spectral data fusion, near-infrared spectroscopy (NIR), Salvia miltiorrhiza, variable selection

## Abstract

Salvia miltiorrhiza: is a widely used Chinese medicinal herb whose quality is significantly influenced by geographical origin. Establishing reliable methods for origin identification is therefore crucial for quality assurance. In this study, 67 batches of *Salvia miltiorrhiza* samples from Shandong, Shanxi, Henan, and Sichuan provinces were analyzed using near-infrared (NIR) and mid-infrared (MIR) spectroscopy combined with chemometric techniques. Six preprocessing methods were applied to optimize spectral data, and PLS-DA models were constructed based on the optimized results. To further improve model performance, uninformative variable elimination (UVE), competitive adaptive reweighted sampling (CARS), and random forest (RF) were employed for variable selection. Discriminant models were then established using NIR, MIR, and fused (NIR + MIR) data, with performance evaluated by accuracy. Results showed that in NIR, the 2nd-RF-PLS-DA model achieved the best performance with 96.72% accuracy, while in MIR, the SG-UVE-PLS-DA model reached 98.33% accuracy. After integrating NIR and MIR data, the 2nd-UVE-PLS-DA model achieved 100% accuracy, demonstrating the strongest discriminative capability. These findings demonstrate that combining NIR and MIR spectroscopy with appropriate preprocessing and variable selection strategies fully exploits complementary spectral information, enabling the construction of rapid, reliable, and efficient discriminant models. This approach provides an effective tool for origin tracing of *Salvia miltiorrhiza* and serves as a methodological reference for advancing quality evaluation of other Chinese herbal medicines.

## 1 Introduction

*Salvia miltiorrhiza* Bge. (Danshen) is a perennial herbaceous plant of the Lamiaceae family, with its dried roots and rhizomes widely used in medicine. With a long history of therapeutic application, it was first documented in the *Shennong Bencao Jing* (*Divine Farmer’s Classic of Materia Medica*) and has since been continuously recorded in successive herbal monographs (Chien et al., 2016). It remains one of the most widely used bulk crude drugs in traditional Chinese medicine (TCM). Modern pharmacological studies have revealed that *Salvia miltiorrhiza* contains two major groups of bioactive constituents, namely, phenolic acids and tanshinones (Liu et al., 2018), which exhibit a broad spectrum of biological activities, including anti-myocardial ischemia, anti-inflammatory, antioxidant, anti-tumor, and hepatoprotective effects (Huang et al., 2024; Jiang et al., 2014; Li et al., 2024; Yang et al., 2025; Zhou et al., 2020). At present, *Salvia miltiorrhiza* is extensively cultivated in major producing regions of China, including Shandong, Shanxi, Henan, and Sichuan (Dai et al., 2024; Li et al., 2022). Previous studies have demonstrated that the production environment exerts a significant influence on the quality of Danshen. Ecological factors such as soil physicochemical properties, climatic conditions, and water quality can modulate secondary metabolic processes, thereby affecting the biosynthesis and accumulation of active compounds (Yu et al., 2025). Consequently, notable differences exist in chemical composition and pharmacological effects among materials of different geographical origins (He et al., 2023). For example, Danshen from Shandong generally contains higher levels of tanshinones, whereas that from Sichuan is richer in salvianolic acid B (Yao et al., 2022). Such regional variation not only compromises the consistency of clinical efficacy but also imposes greater demands on the circulation, quality assessment, and standardization of Danshen materials. Therefore, establishing accurate and rapid methods for origin identification is of great importance to ensure consistency in quality and therapeutic effectiveness.

Currently, the identification and quality evaluation of *Salvia miltiorrhiza* primarily rely on conventional approaches, including morphological observation, microscopic identification, and chromatographic techniques such as high-performance liquid chromatography (HPLC) and gas chromatography–mass spectrometry (GC-MS). Although these methods provide high sensitivity and accuracy, they suffer from inherent limitations, such as dependence on expertise, complicated operation, time-consuming sample pretreatment, high analytical costs, and destructive measurement of samples. As a result, they fall short of meeting the practical requirements for rapid and non-destructive testing. In recent years, advances in spectroscopic techniques have opened new avenues for tracing the geographical origin of TCMs. Among these, Fourier Transform Near-Infrared Spectroscopy (FT-NIR) and Mid-Infrared Spectroscopy (FT-MIR) have attracted wide attention due to their remarkable advantages, including rapidity, non-destructive nature, high throughput, and minimal sample preparation (Li et al, 2025a). These techniques have been extensively applied in fields such as food safety inspection, agricultural product quality control, and authentication of herbal medicines, and they hold considerable promise for provenance identification (Alvarez et al., 2020; Miaw et al., 2018) and quality evaluation of TCMs. NIR spectroscopy is based on overtone and combination vibrations of hydrogen-containing groups within molecules, providing holistic information on the molecular composition of herbs (Okere et al., 2022). In contrast, MIR spectroscopy corresponds to the fundamental vibrations of molecular bonds, offering distinct absorption peaks of functional groups and thus more detailed structural information (Giorgini et al., 2023). When combined, NIR and MIR provide complementary information, greatly enhancing the discriminative capacity of analytical models and effectively improving their accuracy, robustness, and general applicability (Li et al., 2018).

Nevertheless, raw spectral data often suffer from interference factors, including peak overlap, baseline drift, light scattering effects, and background noise, necessitating optimization through chemometric methods (Wang et al., 2025; Zou et al., 2023). Appropriate preprocessing techniques can reduce interference and emphasize meaningful signals, while pattern recognition methods enable effective classification of samples from different origins. For example, Principal Component Analysis (PCA), a commonly used unsupervised approach, extracts the major features of data through dimensionality reduction. Partial Least Squares Discriminant Analysis (PLS-DA), a supervised method, classifies samples by constructing regression models between spectral variables and categorical outcomes (Li et al., 2023). However, the high dimensionality of spectral data and the abundance of variables often lead to redundant information and non-informative wavelengths, which may cause model overfitting or classification bias. Therefore, the incorporation of variable selection strategies is essential to enhance model reliability and predictive performance (Wang et al., 2025).

In this study, NIR and MIR spectroscopy were integrated with multiple chemometric approaches to systematically analyze 67 batches of *Salvia miltiorrhiza* samples collected from four principal production regions, namely, Shandong, Shanxi, Henan, and Sichuan. Six preprocessing techniques, including multivariate scatter correction (MSC), standard normal variate (SNV), mean centering (MC), Savitzky–Golay smoothing (SG), first-order derivative (1st), and second-order derivative (2nd), were employed to optimize the raw spectra by eliminating baseline drift and noise. In addition, three variable selection algorithms, namely, uninformative variable elimination (UVE), competitive adaptive reweighted sampling (CARS), and random forest (RF), were utilized to extract key wavelengths and remove redundant variables. By adopting this systematic strategy, a high-performance and robust PLS-DA model was successfully constructed, which enabled rapid and accurate discrimination of Danshen samples from different geographical origins. This work not only provides valuable insights for improving the quality evaluation and standardization of *Salvia miltiorrhiza* but also offers a methodological reference for origin authentication and quality control of other traditional Chinese medicinal materials.

## 2 Materials and methods

### 2.1 Experimental instruments and materials

#### 2.1.1 Instruments

The equipment used included a TENSOR II Fourier Transform Infrared (FTIR) spectrometer (Bruker, Germany), an ANTARIS II Fourier Transform Near-Infrared (FT-NIR) spectrometer (Thermo Fisher Scientific, United States), an FW-100 high-speed universal pulveriser (Beijing Kewei Yongxing Instrument Co., Ltd., China), an FW-4A powder tablet press (Tianjin Topology Instrument Co., Ltd., China), a DHG-9146A electric heating constant-temperature forced-air drying oven (Shanghai Xiren Scientific Instrument Co., Ltd., China), an MS105DU analytical balance with 0.0001 g precision (Mettler-Toledo, Shanghai, China), and an FW-500 ultrasonic cleaner (Kunshan Ultrasonic Instrument Co., Ltd., China).

#### 2.1.2 Materials

Potassium bromide (spectroscopy grade, 99%, IR grade; Tianjin Kemiou Chemical Reagents Co., Ltd., China). *Salvia miltiorrhiza* (Danshen) decoction pieces were collected in 2025 from different production regions (detailed batch and origin information is provided in Table 1). All samples were authenticated as the dried roots and rhizomes of *Salvia miltiorrhiza* Bge. (Lamiaceae) by Shi Junhan, Deputy Chief Pharmacist at the First Affiliated Hospital of Henan University of Chinese Medicine.

**TABLE 1:** Information on *Salvia miltiorrhiza* samples from different geographical origins.

Code	Origin	Code	Origin	Code	Origin
HN1	Luoyang, Henan	SD8	Pingyi, Shandong	SX12	Wanrong, Shanxi
HN2	Luoyang, Henan	SD9	Linyi, Shandong	SX13	Wanrong, Shanxi
HN3	Luoyang, Henan	SD10	Linyi, Shandong	SX14	Wanrong, Shanxi
HN4	Yuzhou, Henan	SD11	Linyi, Shandong	SX15	Wanrong, Shanxi
HN5	Yuzhou, Henan	SD12	Mengyin, Shandong	SX16	Wanrong, Shanxi
HN6	Jiyuan, Henan	SD13	Ju County, Shandong	SC1	Deyang, Sichuan
HN7	Yuzhou, Henan	SD14	Weifang, Shandong	SC2	Deyang, Sichuan
HN8	Yuzhou, Henan	SD15	Ju County, Shandong	SC3	Deyang, Sichuan
HN9	Yuzhou, Henan	SD16	Linyi, Shandong	SC4	Deyang, Sichuan
HN10	Yuzhou, Henan	SD17	Linyi, Shandong	SC5	Deyang, Sichuan
HN11	Sanmenxia, Henan	SD18	Linyi, Shandong	SC6	Deyang, Sichuan
HN12	Wen County, Henan	SD19	Linyi, Shandong	SC7	Deyang, Sichuan
HN13	Luoyang, Henan	SX1	Wanrong, Shanxi	SC8	Deyang, Sichuan
HN14	Sanmenxia, Henan	SX2	Wanrong, Shanxi	SC9	Deyang, Sichuan
HN15	Sanmenxia, Henan	SX3	Wanrong, Shanxi	SC10	Deyang, Sichuan
HN16	Yuzhou, Henan	SX4	Wanrong, Shanxi	SC11	Deyang, Sichuan
SD1	Linyi, Shandong	SX5	Wanrong, Shanxi	SC12	Deyang, Sichuan
SD2	Rizhao, Shandong	SX6	Wanrong, Shanxi	SC13	Deyang, Sichuan
SD3	Rizhao, Shandong	SX7	Wanrong, Shanxi	SC14	Deyang, Sichuan
SD4	Heze, Shandong	SX8	Wanrong, Shanxi	SC15	Deyang, Sichuan
SD5	Ju County, Shandong	SX9	Wanrong, Shanxi	SC16	Deyang, Sichuan
SD6	Linyi, Shandong	SX10	Wanrong, Shanxi	​	​
SD7	Linyi, Shandong	SX11	Wanrong, Shanxi	​	​

Henan (16 batches), Shandong (19 batches), Shanxi (16 batches), and Sichuan (16 batches), for a total of 67 batches.

### 2.2 Sample preparation

#### 2.2.1 NIR spectroscopy sample preparation

*Salvia miltiorrhiza* slices were thoroughly dried under low-temperature conditions to prevent decomposition or loss of active constituents caused by excessive heat. The dried samples were ground using a grinder and passed through a 50-mesh sieve to ensure uniform particle size distribution and to minimize the particle size effect on NIR spectra. Approximately 6 g of *Salvia miltiorrhiza* powder were accurately weighed and stored in a sealed bag for later use.

#### 2.2.2 MIR spectroscopy sample preparation

*Salvia miltiorrhiza* slices were likewise dried and ground under low-temperature conditions and then passed through a 200-mesh sieve to ensure sufficient fineness, thereby improving the homogeneity of mixing with KBr and enhancing infrared transmittance. Exactly 1 mg of *Salvia miltiorrhiza* powder was accurately weighed and mixed with 200 mg of spectroscopy-grade KBr. The mixture was thoroughly ground in an agate mortar and dried under an infrared lamp to remove moisture, thus preventing interference from water absorption peaks. The well-mixed powder was placed into a dedicated tableting mould and pressed at 6 MPa for 40 s to form a uniform, translucent disc for MIR transmission spectroscopy.

### 2.3 Test methods

#### 2.3.1 NIR spectroscopy testing method

Solid diffuse reflectance sampling was performed using a Fourier transform near-infrared (FT-NIR) spectrometer equipped with an integrating sphere accessory to ensure uniform and stable optical signals. The laboratory environment during NIR acquisition was strictly controlled at 20 °C–25 °C and 25%–35% relative humidity to ensure measurement stability. The scanning wavenumber range was set to 10,000–4,000 cm^-1^, covering the major characteristic absorption bands of the NIR region. Each sample was scanned 64 times at a spectral resolution of 8 cm^-1^, ensuring a balance between the signal-to-noise ratio and spectral resolution. A background spectrum was recorded before each batch of spectral acquisition for blank subtraction to minimize interference from the instrument and the environment. During spectral collection, the measurement order of all samples was randomized to avoid possible sequence effects and instrumental drift. During each measurement, the quartz sample cup was gently shaken to ensure a uniform and flat powder surface, thereby reducing light-scattering effects caused by uneven particle distribution. Each sample was independently scanned three times, and the averaged spectra were used as the final raw data to ensure representativeness and reproducibility.

#### 2.3.2 MIR spectroscopy testing method

MIR spectra were obtained using the KBr pellet method and recorded in transmission mode with a Fourier transform mid-infrared (FT-MIR) spectrometer. The scanning wavenumber range was set to 4,000–400 cm^-1^, covering fundamental vibrational regions of molecular functional groups. Each spectrum was scanned 16 times with a spectral resolution of 4 cm^-1^ to ensure clear absorption peaks within a reasonable measurement time, and the scanning speed was set to 0.2 cm s^-1^. To minimize environmental interference, MIR acquisition was performed under controlled laboratory conditions (temperature 20 °C–25 °C; relative humidity 25%–35%), and the instrument automatically subtracted CO_2_ and H_2_O absorption bands in real time. Before testing, the KBr pellets were examined for uniformity and transparency to ensure sufficient light transmittance. All samples were measured in a randomized order to reduce systematic bias and minimize the impact of instrument drift. Each sample was independently scanned three times, and the averaged spectrum was used as the raw data to enhance stability and comparability.

### 2.4 Methodological validation

To ensure the reliability of the spectral data and the scientific validity of the methods, repeatability, within-laboratory reproducibility, and stability tests were conducted for both NIR and MIR spectroscopy.

#### 2.4.1 Validation of the NIR spectroscopy method

HN1 powder samples prepared according to the procedure described in Section 2.2.1 were measured six consecutive times under identical conditions to evaluate instrument repeatability. To examine method within-laboratory reproducibility, six independent HN1 powder samples were prepared and measured separately, and their spectral consistency was compared. Furthermore, to assess sample stability within a defined time frame, the same HN1 powder sample was repeatedly measured at 30-min intervals, and the consistency of the resulting spectra was analyzed.

#### 2.4.2 Validation of the MIR spectroscopy method

HN1 pellets prepared following the method described in Section 2.2.2 were measured six consecutive times under identical conditions to assess instrument repeatability. To evaluate method within-laboratory reproducibility, six separate pellets were independently prepared from the HN1 powder sample and scanned individually, with the spectral results compared. Finally, pellets prepared from the same HN1 powder sample were measured at different time points (every 30 min). Sample stability during the testing process was assessed by comparing spectral variations over time.

### 2.5 Data analysis

Spectral data were preprocessed using MATLAB R2022b, and PCA was carried out with SIMCA 14.1 software. PLS-DA was performed with the built-in Classification 6.0 toolbox in MATLAB R2022b, and the classification performance was evaluated based on the accuracy obtained through leave-one-out cross-validation (LOOCV). During spectral preprocessing, six methods were applied, including MSC, SNV, MC, SG, 1st derivative, and 2nd derivative. These preprocessing techniques reduced interference caused by particle effects and scattering variations in the spectral data, minimized baseline drift and noise, and enhanced the resolution of characteristic absorption peaks. For variable selection, three algorithms were employed, namely, UVE, CARS, and RF. The UVE method removes noise variables that are irrelevant to classification, CARS identifies the optimal combination of spectral bands through adaptive sampling, and RF variable importance analysis recognizes feature wavelengths that contribute significantly to classification from a nonlinear perspective.

## 3 Results

### 3.1 Method validation results

#### 3.1.1 NIR spectroscopy method validation results

In the validation of NIR, the relative standard deviations (RSD) for repeatability, within-laboratory reproducibility, and stability tests were 0.015%, 0.045%, and 0.015%, respectively. The results indicate that the method exhibits excellent instrument repeatability and within-laboratory reproducibility, and the samples maintained good stability during the testing process.

#### 3.1.2 MIR spectroscopy method validation results

In the validation of MIR, the RSD values for repeatability, within-laboratory reproducibility, and stability tests were 0.007%, 0.015%, and 0.021%, respectively. The results confirm that the method provides good repeatability and within-laboratory reproducibility, and the samples displayed high stability throughout testing.

Taken together, both NIR and MIR spectroscopy methods achieved high levels of repeatability, within-laboratory reproducibility, and stability. These findings demonstrate that the methods fully satisfy experimental requirements and establish a reliable foundation for subsequent modeling and data analysis.

### 3.2 Raw infrared spectra acquisition results

**FIGURE 1:**
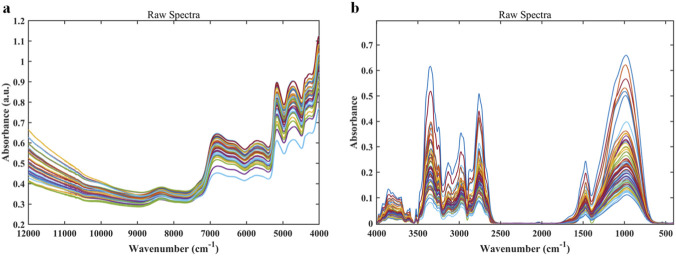
**(a)** Raw near-infrared (NIR) spectra; **(b)** raw mid-infrared (MIR) spectra.

Figure 1a shows the NIR raw spectrum of the *Salvia miltiorrhiza* sample. The overall spectrum exhibits typical absorption characteristics of organic compounds. A prominent absorption peak appears near 6,900 cm^-1^, which is mainly attributed to the first overtone absorption of hydroxyl (O–H) stretching vibrations. The absorption peak near 5,060 cm^-1^ corresponds to the combination vibration of C–H bonds, and the minor peak around 4,600 cm^-1^ is likely associated with the combination band of C–H bonds. These absorption features reflect the fundamental molecular vibration information of alcohols, phenols, and organic acids contained in the *Salvia miltiorrhiza* sample.

Figure 1b presents the raw MIR spectrum of the *Salvia miltiorrhiza* sample. The strong absorption peak at 3,500–3,200 cm^-1^ corresponds to O–H stretching vibrations, which may originate from water, phenolic acids, and polysaccharides. The peaks at 2,920 cm^-1^ and 2,850 cm^-1^ represent typical C–H stretching vibrations (CH_2_, CH_3_), mainly arising from tanshinones and organic acids. The strong absorption near 1,650 cm^-1^ is attributed to the C=O stretching vibration of the phenanthrene quinone structure in tanshinones, serving as a key characteristic signal of the active components in *Salvia miltiorrhiza*. Absorption peaks around 1,510 cm^-1^ and 1,600 cm^-1^ are related to aromatic ring skeletal vibrations, indicating the presence of salvianolic acids and flavonoids. The multiple absorption peaks between 1,450 and 1,000 cm^-1^ are mainly assigned to C–H bending vibrations and C–O stretching vibrations, together reflecting the complex molecular structure of *Salvia miltiorrhiza*.

Overall, the NIR and MIR spectra of *Salvia miltiorrhiza* samples from different geographical origins exhibit similar overall profiles. The positions of the major absorption peaks are largely consistent, with extensive overlap of spectral lines, particularly in key characteristic absorption regions where differences are subtle. Therefore, visual inspection of raw spectra alone is insufficient for effective origin discrimination. Further spectral preprocessing is required to reduce background interference and enhance characteristic differences, in combination with chemometric analysis.

### 3.3 Spectral preprocessing analysis

#### 3.3.1 NIR spectral preprocessing analysis

**FIGURE 2:**
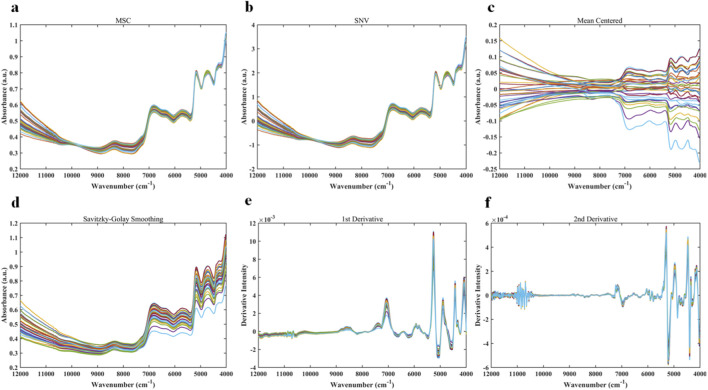
Preprocessed NIR spectra: **(a)** MSC; **(b)** SNV; **(c)** MC; **(d)** SG; **(e)** 1st derivative; **(f)** 2nd derivative.

The results of NIR spectra after preprocessing with six different methods are shown in Figures 2a–f. These figures correspond to spectra obtained from raw near-infrared data after applying MSC, SNV, MC, SG, 1st derivative, and 2nd derivative, respectively. MSC and SNV were mainly used to reduce baseline drift caused by particle effects and scattering variations (Hu et al., 2021). MC helped minimize the influence of sample concentration differences, while SG smoothing reduced random noise (Zhao et al., 2023). The 1st and 2nd derivative preprocessing enhanced weak absorption peaks and improved spectral resolution. Although multiple preprocessing methods were applied, the overall spectral curves still showed a strong tendency to overlap. Samples from different geographical origins did not visually form distinct clusters, making it difficult to differentiate *Salvia miltiorrhiza* samples by origin. This observation suggests that even with optimized treatments such as baseline correction and scatter correction, visual inspection of spectra alone is insufficient for effective origin discrimination. Chemometric approaches are therefore required for feature extraction and pattern recognition.

#### 3.3.2 MIR spectral preprocessing analysis

**FIGURE 3:**
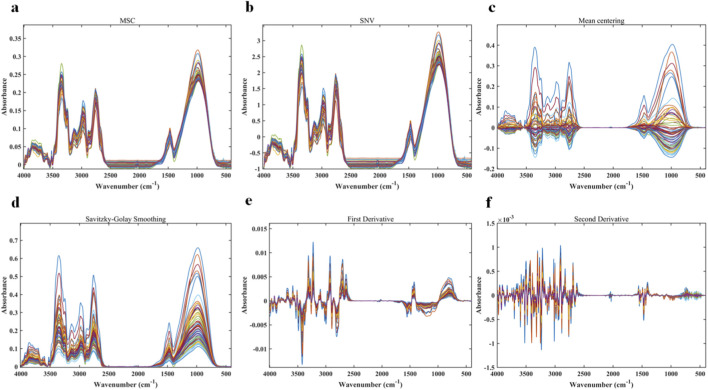
Preprocessed MIR spectra: **(a)** MSC; **(b)** SNV; **(c)** MC; **(d)** SG; **(e)** 1st derivative; **(f)** 2nd derivative.

The results of MIR spectra after preprocessing with six different methods are shown in Figures 3a–f. These figures correspond to spectra obtained from raw MIR data after applying MSC, SNV, MC, SG, 1st derivative, and 2nd derivative, respectively. Overall, the preprocessed spectral curves still exhibited a high degree of overlap, and the characteristic peaks of *Salvia miltiorrhiza* samples from different origins could not be clearly distinguished by visual inspection. Although preprocessing improved the signal-to-noise ratio and enhanced peak clarity to some extent, it did not substantially increase the visual discriminability of origin differences.

These findings suggest that the inherent spectral differences between NIR and MIR data are largely masked by complex baseline effects and overlapping multicomponent peaks. Reliable origin discrimination cannot be achieved solely through spectral preprocessing and direct visual comparison. This further highlights the necessity of employing chemometric modeling approaches, which provide the basis for subsequent variable selection and classification modeling.

### 3.4 Principal Component Analysis

**FIGURE 4:**
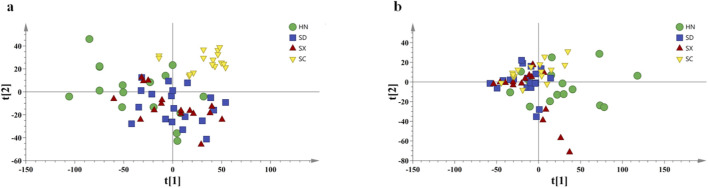
PCA score plots based on raw spectra: **(a)** NIR; **(b)** MIR.

In the PCA score plot of NIR spectra shown in Figure 4a, sample points are distributed within the principal component plane defined by PC1 and PC2, displaying a pattern of clustered aggregation accompanied by partial dispersion. PC1 and PC2 contributed 71.60% and 24.00% of the variance, respectively, with a cumulative contribution of 95.60%. This demonstrates that the first two principal components effectively capture the majority of variation in the original NIR spectral data. Most samples are concentrated around the center of the coordinate axes, forming a band-like distribution with noticeable width, and SC samples exhibit a certain degree of aggregation. Overall, samples from different origins display high similarity in their NIR spectral information. A few outliers deviate from the main cluster and are located at the periphery, which may result from spectral differences associated with storage conditions, moisture content, or batch variations. Although some spatial distribution differences can be observed, the extensive overlap between categories prevents the establishment of clear origin-discriminating boundaries. These results suggest that although NIR spectra are sensitive to hydrogen-containing functional groups, their overall variability is insufficient for significantly distinguishing sample origins under unsupervised modeling.

The PCA score plot of MIR spectra in Figure 4b shows a more compact distribution pattern, with sample points more densely clustered in the principal component space compared with NIR. This indicates that the functional group vibration information captured by MIR spectra exhibits relatively smaller variation among samples from different origins. PC1 and PC2 of the MIR spectra contributed 69.30% and 24.30% of the variance, respectively, with a cumulative contribution of 93.60%, demonstrating that the first two components account for most of the original variability. The majority of samples are concentrated in the central region, with relatively few outliers at the periphery, further indicating high similarity among samples from different origins. Although certain samples show a tendency to scatter along the two principal component directions (PC1 and PC2), no distinct clusters separated by origin are formed. Instead, most samples remain centered and overlapping, suggesting that MIR spectral information alone is also insufficient for effective origin discrimination.

Supplementary Figures 1a–1f present PCA score plots of NIR spectra after optimization using six different preprocessing methods. Compared with the original PCA results, the preprocessed plots exhibit minor improvements, yet extensive overlapping persists. Supplementary Figuress 2a–2f show PCA score plots of MIR spectra after similar preprocessing. Severe sample overlap remains, making origin-based separation unachievable. Taken together, these results indicate that even with optimized preprocessing, PCA models alone cannot reliably identify the geographical origin of *Salvia miltiorrhiza*.

### 3.5 PLS-DA analysis of different preprocessing methods

#### 3.5.1 NIR PLS-DA analysis

**FIGURE 5:**
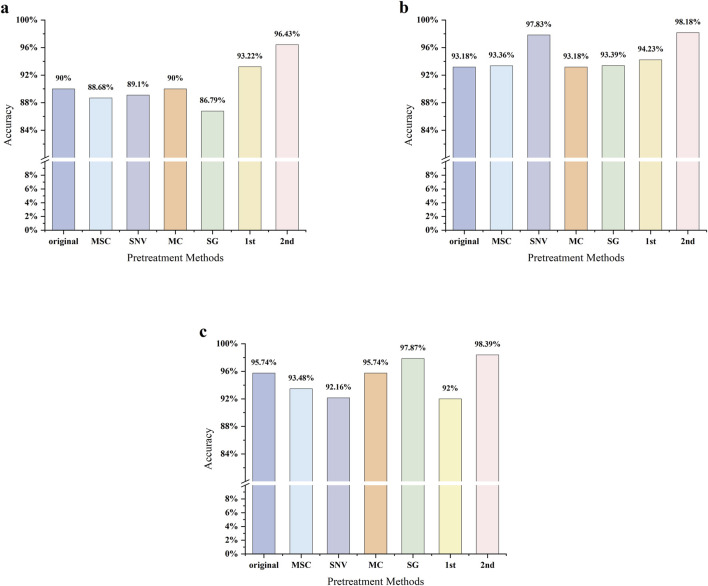
Accuracies of PLS-DA models under different preprocessing methods: **(a)** NIR spectra; **(b)** MIR spectra; **(c)** combined NIR–MIR spectra.

The accuracy results of the NIR PLS-DA model are presented in Figure 5a. The model constructed using raw NIR spectra achieved an accuracy of 90.00%, indicating that unprocessed spectral information already allows reasonably accurate classification of most samples. Among the different preprocessing methods, MC yielded results essentially identical to those of the raw data, also achieving an accuracy of 90.00%. This suggests that while MC helps reduce the effect of random noise, it contributes little to improving overall spectral features.

In contrast, derivative preprocessing substantially improved model performance. The 1st derivative and 2nd derivative increased the accuracy to 93.22% and 97.00%, respectively, representing improvements of 3.22% and 7.00% over the raw spectra. These improvements can be attributed to the ability of derivative processing to effectively eliminate baseline drift, which is common in NIR spectra, and to separate overlapping absorption peaks, thereby enhancing feature information related to chemical composition. These findings demonstrate that derivative-based preprocessing methods provide significant advantages in NIR spectral modeling, particularly for analyzing complex systems.

Not all preprocessing methods improved model performance. After MSC, the accuracy decreased to 88.68%, representing a 1.32% decline relative to the raw spectra. SNV preprocessing similarly reduced performance, yielding an accuracy of 89.10%, a 0.90% decrease. The poorest results were obtained with SG smoothing, which achieved only 86.79%, a 3.21% reduction. This likely resulted from inappropriate SG parameter selection, leading to excessive signal smoothing or the introduction of spurious peaks, which diminished spectral details closely associated with sample differences and weakened the model’s discriminative capability.

Overall, these results highlight that preprocessing strategies exert a substantial influence on the classification performance of NIR PLS-DA models. Derivative methods are particularly effective, as they enhance model discrimination by preserving and amplifying valid spectral information (Wang et al., 2023). Conversely, inappropriate preprocessing methods, such as unsuitable smoothing, may excessively remove valid signals or introduce artifacts, thereby distorting the original spectral profile and causing the loss of critical information such as peak position and width. Thus, the choice of preprocessing methods should be carefully optimized by considering both spectral characteristics and research objectives. Achieving a balance between noise reduction, baseline correction, and information enhancement is essential to avoid additional interference and to ensure the accuracy and reliability of classification models.

#### 3.5.2 MIR PLS-DA analysis

The accuracy results of MIR PLS-DA modeling are shown in Figure 5b. When modeling directly with raw MIR spectra, the model achieved an accuracy of 93.18%, outperforming the raw NIR spectra (90.00%). This indicates that the functional group vibration information captured in MIR spectra provides higher discriminative power for sample classification.

Model performance generally improved after applying different preprocessing methods. MSC and SG preprocessing yielded accuracies of 93.36% and 93.39%, respectively. Although the improvement was limited, these results still suggest a modest optimization effect. 1st derivative preprocessing further increased accuracy to 94.23%, enhancing the model’s discriminative ability by reducing baseline drift and spectral noise.

The most substantial improvements were observed with SNV and 2nd derivative preprocessing. SNV raised the accuracy to 97.83%, demonstrating its effectiveness in correcting multiplicative interference caused by particle size and scattering, thereby clarifying spectral differences associated with chemical composition. The 2nd derivative delivered the best performance, with accuracy reaching 98.18%. In addition to eliminating baseline drift, it sharpened characteristic absorption peaks and separated overlapping peaks, improving spectral resolution and distinctiveness. This provided the model with more discriminative input information.

Overall, the MIR PLS-DA model exhibited high classification accuracy even without preprocessing. However, selecting appropriate preprocessing methods, particularly SNV and 2nd derivatives, further enhanced model performance. These results highlight that combining spectral features with suitable preprocessing strategies is critical for fully exploiting spectral information and improving classification capability. Compared to NIR models, MIR models demonstrated superior overall performance, indicating that MIR spectroscopy holds substantial application potential for discriminating the geographical origin of *Salvia miltiorrhiza*.

#### 3.5.3 NIR–MIR fusion PLS-DA analysis

The accuracy results of PLS-DA modeling based on NIR–MIR fusion spectra are shown in Figure 5c. The model constructed using raw fused spectra achieved an accuracy of 95.74%, which was higher than the models built with raw NIR (90.00%) or MIR (93.18%) spectra alone. This demonstrates that spectral fusion can effectively integrate complementary information from both spectral regions, thereby enhancing overall discriminative capability. Model performance varied depending on the preprocessing method applied. MSC reduced the accuracy to 93.48%, while SNV further decreased it to 92.16%. 1st derivative preprocessing also yielded suboptimal results, with accuracy dropping to 92.00%. These findings suggest that for complex fused datasets, some commonly applied preprocessing techniques may not provide the expected optimization. Instead, due to inappropriate parameter selection or limited applicability, they may cause information loss or amplify noise, ultimately reducing model performance. MC preprocessing produced results identical to the raw fused data, maintaining an accuracy of 95.74%, indicating only a limited effect on fused spectra.

In contrast, SG smoothing and 2nd derivative preprocessing achieved superior performance, with accuracies increasing to 97.87% and 98.39%, respectively. SG smoothing effectively reduced high-frequency noise in the fused spectra while preserving the overall spectral profile, thereby enhancing model stability. The 2nd derivative provided the greatest improvement by eliminating baseline drift, sharpening characteristic absorption peaks, and separating overlapping peaks. This enabled more comprehensive extraction of critical information from the fused data, significantly improving spectral discriminative power.

Overall, NIR–MIR spectral fusion effectively integrates complementary chemical information from both spectral regions and, when paired with appropriate preprocessing strategies, can substantially enhance model classification accuracy. Particularly when combined with SG smoothing or 2nd derivative preprocessing, the potential of fused spectra is maximized, enabling the construction of more robust and highly accurate discriminant models. These results confirm that spectral fusion is an effective strategy for supporting the reliable origin identification of complex samples.

#### 3.5.4 Comprehensive evaluation of separate and fusion modeling using NIR and MIR spectra

The modeling results of *Salvia miltiorrhiza* spectral data indicate that both NIR and MIR spectra exhibit strong discriminative capability in PLS-DA analysis, although their performance and stability differ. NIR spectra showed substantial improvement with derivative preprocessing, achieving a maximum accuracy of 97.00%, but the results were highly sensitive to preprocessing strategies. By contrast, MIR spectra displayed a higher baseline accuracy (93.18%) and achieved superior performance under SNV and 2nd derivative preprocessing (up to 98.18%), reflecting greater robustness and stronger interpretability.

When NIR and MIR spectra were fused, model performance improved further. The raw fusion spectra achieved an accuracy of 95.74%, which was clearly higher than either spectrum alone. Under 2nd derivative preprocessing, the fusion model reached 98.39%, surpassing the best results obtained with standalone NIR or MIR, and representing the highest accuracy among all model combinations. Importantly, fusion also reduced the model’s dependence on individual preprocessing methods. For example, while SG preprocessing caused a severe decline in NIR accuracy (86.79%), the same method maintained a high accuracy of 97.87% with fused spectra, demonstrating improved stability and resistance to noise or interference.

To further elaborate, spectral fusion provides advantages that extend beyond numerical improvements. Fusion enhances model robustness by combining the complementary chemical information contained in NIR and MIR. NIR primarily captures broad overtone and combination vibrations associated with physical attributes of the samples, while MIR provides sharper and more distinct absorptions corresponding to fundamental molecular vibrations. Integrating these two forms of information produces a more complete representation of the chemical characteristics of the samples. Fusion also improves prediction stability by reducing the influence of fluctuations caused by sample heterogeneity, environmental conditions, and preprocessing variations. Moreover, fusion reduces interference effects because the model does not rely heavily on any single set of wavelengths. Instead, the discriminative information is distributed across multiple chemically meaningful regions, which helps buffer the model against individual disturbances.

Overall, NIR and MIR spectra each provide distinct advantages for geographical origin discrimination. The fusion strategy maximizes the integration of their complementary chemical information, compensates for the shortcomings of single-spectrum approaches, and enhances both robustness and classification accuracy. Although the numerical improvements appear modest, they correspond to clearer classification boundaries and more reliable discrimination results, which are of significant importance for achieving precise origin identification of *Salvia miltiorrhiza* samples.

### 3.6 Variable selection combined with PLS-DA modeling

#### 3.6.1 Analysis of variable counts across different selection methods

**FIGURE 6:**
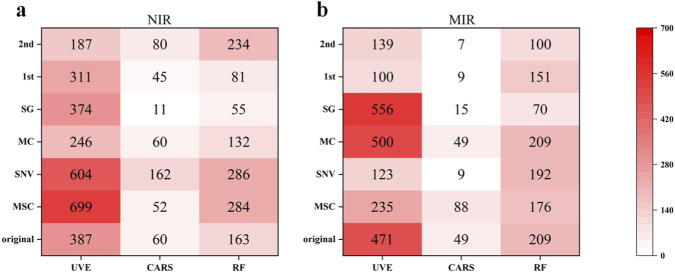
Number of selected variables by different methods: **(a)** NIR spectra; **(b)** MIR spectra.

Figures 6a,b present the number of feature variables obtained after variable selection for NIR and MIR spectra, respectively. The results indicate that the CARS method consistently demonstrates strong dimensionality reduction capability across both raw and preprocessed spectra, selecting only a very limited number of feature wavelengths. By contrast, the UVE method tends to produce a considerably larger set of variables.

For NIR data, MSC preprocessing combined with UVE resulted in the largest number of variables, reaching 699, followed by the SNV-UVE combination with 604 variables. In contrast, the SG-CARS combination produced the fewest variables, selecting only 11. For MIR data, the SG-UVE combination yielded the greatest number of variables (556), whereas the combinations of SNV, 1st derivative, and 2nd derivative preprocessing with CARS dramatically reduced the number of variables to single digits. Notably, the 2nd-CARS approach selected as few as 7 variables.

These findings highlight the significant influence of both variable selection methods and preprocessing strategies on the number of feature wavelengths extracted. The CARS method is highly effective in extracting the most informative features across different spectral regions, thereby enabling the construction of extremely simplified models (Kamruzzaman et al., 2022). In contrast, UVE generally retains a broader range of spectral information. Moreover, the variations observed across preprocessing approaches indicate distinct sensitivities in variable selection outcomes. Collectively, these results provide a valuable reference for balancing model complexity with performance optimization in spectral analysis.

#### 3.6.2 UVE-PLS-DA classification results

**FIGURE 7:**
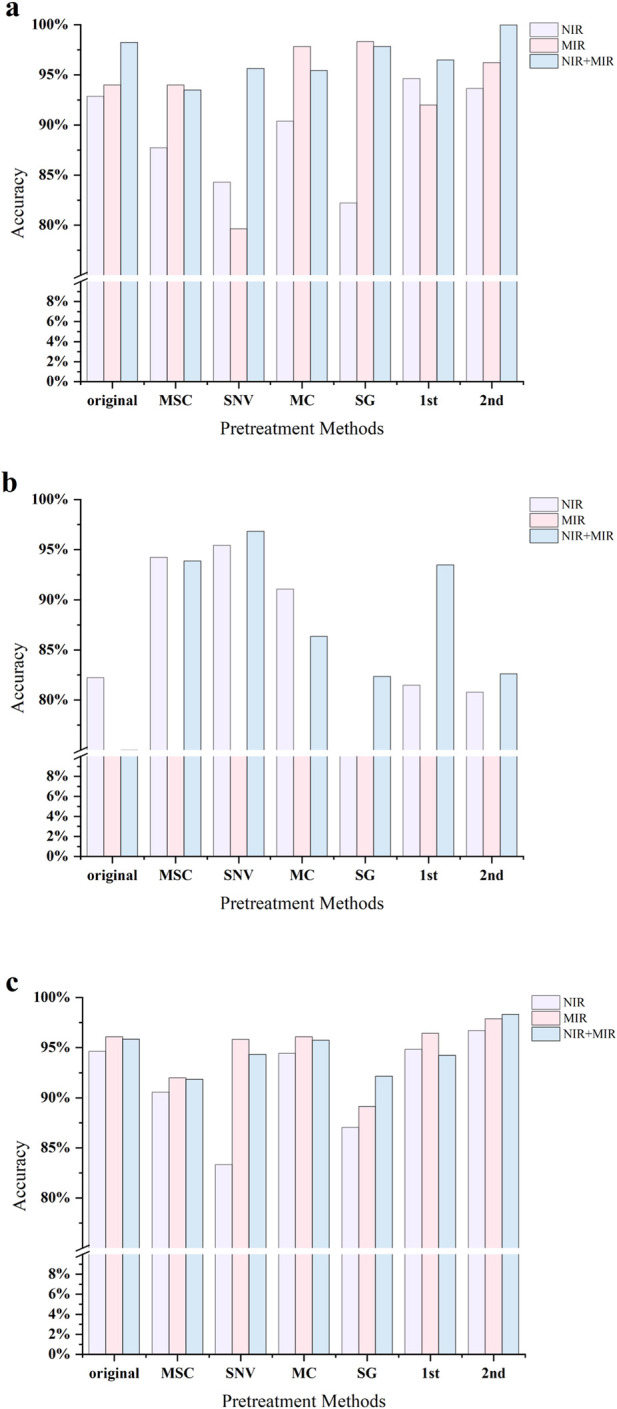
Accuracies of PLS-DA models with variable selection under different preprocessing methods: **(a)** UVE; **(b)** CARS; **(c)** RF.

The classification results after UVE variable selection are shown in Figure 7a. UVE-PLS-DA demonstrated consistently strong discrimination performance across different spectral datasets. For NIR spectra, UVE screening increased the model accuracy to 92.86%, representing a 2.86% improvement over the raw NIR spectral model (90.00%). When combined with 1st derivative preprocessing, accuracy further improved to 94.64%, indicating that 1st derivative processing effectively reduced baseline drift and spectral noise, thereby enhancing the correlation between spectral features and class labels and improving classification performance.

For MIR spectra, UVE also produced clear benefits. Accuracy increased to 94.00% after UVE selection, representing a modest improvement of 0.82% compared with the raw MIR model (93.18%). Among different preprocessing methods, the SG-UVE combination yielded the best results, achieving 98.33% accuracy, a substantial 4.33% increase relative to the raw UVE model. This suggests that SG smoothing effectively suppresses spectral noise in MIR data and works synergistically with UVE’s feature extraction capability, significantly enhancing discriminative power.

For fused spectral data, UVE again exhibited excellent performance. Applying UVE to the raw fused spectra increased accuracy to 98.25%, 2.51% higher than the raw fusion model (95.74%). When further combined with 2nd derivative preprocessing, the 2nd-UVE-PLS-DA model achieved 100% accuracy, representing the highest performance among all method combinations evaluated in this study. This demonstrates that UVE effectively extracts key feature variables under multi-source spectral fusion conditions, maximizing the retention of information related to sample chemical composition differences, while ultimately constructing the highest-performing discriminant model.

#### 3.6.3 CARS-PLS-DA classification results

The classification results after CARS variable selection are shown in Figure 7b. For NIR spectra, CARS screening resulted in a model accuracy of 82.22%, representing a 7.78% decrease compared with the raw NIR model (90.00%). This suggests that without appropriate preprocessing, CARS may overly compress the variable space, leading to the exclusion of critical wavelengths closely associated with sample differences. After MSC preprocessing, accuracy improved substantially, with the MSC-CARS-PLS-DA model achieving 94.23%, indicating that MSC effectively mitigates scattering effects and enhances the feature extraction capability of CARS. These findings highlight that the performance of CARS is highly sensitive to preprocessing, and its dimensionality reduction advantage can only be fully realized when combined with suitable preprocessing methods.

In MIR spectra, however, CARS performed poorly. The raw MIR spectrum combined with CARS achieved an accuracy of only 62.96%, a substantial 30.22% decrease compared with the raw MIR model (93.18%). Following SNV preprocessing, accuracy further declined to 45.45%, underscoring that CARS is particularly vulnerable to high-frequency noise and background interference in MIR spectra. These results demonstrate that the complex background and noise characteristics in MIR data strongly affect CARS, causing it to misinterpret valid spectral information as redundant variables and leading to severe deterioration in model performance.

For fused spectral data, CARS again yielded unsatisfactory results, with the raw fused model achieving 75.00% accuracy after CARS screening, a decrease of 20.74% compared with the raw fusion model (95.74%). However, when combined with SNV preprocessing, the SNV-CARS-PLS-DA model achieved a markedly improved accuracy of 96.83%, approaching the performance levels obtained with UVE and RF. These results suggest that although CARS alone may lead to severe performance degradation due to its sensitivity to noise and background interference, the complementary nature of multi-source spectral fusion can partially compensate for these limitations, enabling dimensionality reduction while preserving critical discriminative information.

#### 3.6.4 RF-PLS-DA classification results

The classification results after RF variable screening are shown in Figure 7c. RF-PLS-DA exhibited stable and excellent performance across different spectral datasets. In NIR spectra, the RF-screened model achieved an accuracy of 94.64%, representing a 4.64% improvement over the original NIR model. With 2nd derivative preprocessing, the 2nd-RF-PLS-DA model further improved to 96.72%, indicating that RF effectively captures derivative-enhanced spectral information and thereby strengthens classification performance. In MIR spectra, RF also performed exceptionally well. The RF-screened model achieved 96.08% accuracy, 2.90% higher than the raw MIR model, while the 2nd-RF-PLS-DA model reached 97.87%, highlighting RF’s strong ability to identify discriminative MIR wavelengths.

In fused spectral data, the advantages of RF became even more pronounced. The RF-screened model achieved 95.84% accuracy with raw fusion spectra, and performance further increased to 98.33% after 2nd derivative preprocessing. These findings demonstrate that RF not only handles high-dimensional spectral data efficiently but also fully exploits the complementary information between NIR and MIR, thereby ensuring robust and high-level classification performance.

When comparing the three variable selection methods, UVE achieved the best performance in fused data, reaching 98.25% accuracy with raw spectra and attaining 100% after 2nd derivative preprocessing. RF consistently maintained stable, high performance across both single-spectrum and fused datasets. Although CARS showed weaker performance in MIR spectra, its accuracy could be markedly improved through data fusion and suitable preprocessing.

#### 3.6.5 Comprehensive evaluation of three variable selection methods

A systematic comparison was conducted on the discriminative performance of three representative variable selection methods (UVE, CARS, and RF) combined with PLS-DA. Results revealed notable differences among the methods in terms of variable screening capacity, model performance, and dependence on preprocessing strategies. UVE consistently demonstrated outstanding classification performance in both single-spectrum and fused data, achieving 100% accuracy, the best result in this study, when combined with 2nd derivative preprocessing on fused spectra. The advantage of UVE lies in its ability to retain comprehensive spectral information while effectively eliminating redundant variables, thereby enhancing inter-sample differentiation. Its robust feature extraction capability makes it particularly effective in multi-source spectral fusion contexts.

CARS is characterized by its strong dimensionality reduction capability, substantially minimizing variable numbers and enabling the construction of highly simplified models. However, its performance is highly sensitive to spectral quality and preprocessing methods. In NIR data, combining CARS with MSC preprocessing significantly improved accuracy, whereas in MIR data, CARS exhibited pronounced sensitivity to noise and background interference, resulting in performance degradation. Importantly, data fusion combined with appropriate preprocessing (e.g., SNV) partially alleviated these limitations, markedly enhancing classification performance. This suggests that while CARS holds potential for building minimalist models, its application requires careful handling and robust spectral preprocessing strategies.

RF demonstrated stable and consistently high performance across NIR, MIR, and fused datasets. Regardless of the spectral type, RF effectively improved model accuracy and showed strong adaptability to different preprocessing methods. Its ensemble learning mechanism ensures robust feature selection and provides variable importance rankings, offering valuable insights for chemical interpretation and mechanistic analysis. Consequently, RF combines accuracy, stability, and interpretability, highlighting its broad application potential in complex spectral discrimination tasks.

When comparing the performance of the three methods under data fusion, both NIR and MIR fusion models achieved substantial improvements over single-spectrum models. With UVE, the fusion model attained 98.25% accuracy, improving by 5.39% and 4.25% compared to NIR (92.86%) and MIR (94.00%) single-spectrum models, respectively. After the 2nd derivative preprocessing, the 2nd-UVE-PLS-DA model reached a perfect accuracy of 100%, underscoring the effectiveness of the fusion strategy in integrating complementary spectral information and significantly enhancing classification capability. For CARS, the fusion model initially achieved 75.00% accuracy, which was lower than UVE and RF, but accuracy improved substantially to 96.83% after SNV preprocessing. This demonstrates that data fusion, combined with suitable preprocessing, can greatly mitigate the instability and information loss often associated with CARS. With RF, fusion models consistently outperformed single-spectral models, and after 2nd derivative preprocessing, the 2nd-RF-PLS-DA model reached 98.33% accuracy. These findings confirm that fusion strategies coupled with RF selection effectively synergize multi-source spectral information to construct models with superior classification performance.

Overall, the fusion of NIR and MIR spectra fully leveraged their complementary strengths in functional group information and fingerprint region features. By integrating global information across different spectral regions, fusion models significantly enhanced accuracy and robustness, providing a more comprehensive and reliable analytical approach for the precise geographical discrimination of *Salvia miltiorrhiza*.

## 4 Conclusion

In summary, this study developed a method for origin identification of *Salvia miltiorrhiza* based on the fusion of NIR and MIR spectra combined with chemometric modeling. To address challenges such as baseline drift, scattering interference, and overlapping spectral peaks in raw NIR and MIR data, multiple preprocessing and feature selection methods were systematically compared. This strategy effectively enhanced feature signal extraction and markedly improved model discrimination performance. For single-spectrum modeling, the NIR spectrum achieved optimal performance with the 2nd-RF-PLS-DA model, reaching an accuracy of 96.72%. In MIR spectra, the SG-UVE-PLS-DA model yielded the highest accuracy at 98.33%. By integrating NIR and MIR spectra and applying the 2nd-UVE-PLS-DA method, the constructed discriminant model achieved 100% accuracy, enabling highly efficient and precise origin identification of *Salvia miltiorrhiza* and demonstrating clear superiority over single-spectrum models. The proposed combination of spectral fusion and feature selection provides a reliable and efficient technical solution for the geographical tracing of *Salvia miltiorrhiza*, while also serving as a methodological reference for rapid, non-destructive identification and quality control of other Chinese herbal medicines. Future research will focus on incorporating deep learning algorithms and integrating multi-modal quality indicators, such as liquid chromatography and mass spectrometry, to establish a more comprehensive and precise framework for the quality evaluation of Chinese medicinal materials.

## 5 Discussion

### 5.1 Role and selection of preprocessing methods

The results indicate that different preprocessing methods exert a substantial impact on model performance. Depending on their objectives, preprocessing approaches can be grouped into four categories, namely, baseline correction, scatter correction, smoothing, and scaling (Li et al., 2021). 1st and 2nd derivatives are used to eliminate baseline drift, thereby minimizing the influence of instrument background or signal drift (Bandeira et al., 2024). MSC and SNV reduce scattering effects caused by uneven particle distribution and variations in particle size. SG smoothing diminishes random noise, thereby improving the signal-to-noise ratio of spectral data. MC standardizes dimensional scales to prevent adverse effects arising from excessive differences in data magnitude (Zong et al., 2024). Notably, derivative preprocessing, particularly the 2nd derivative, greatly enhanced model accuracy for both NIR and MIR data. This improvement is consistent with its theoretical capacity to eliminate baseline drift, separate overlapping peaks, and sharpen spectral features, thereby enhancing spectral resolution. However, inappropriate preprocessing may compromise classification accuracy, highlighting the necessity of selecting preprocessing strategies tailored to the specific characteristics of the spectra.

### 5.2 Applicability of feature selection methods

Feature selection effectively reduces redundancy, strengthens model robustness, and enhances interpretability. In this study, three methods, UVE, CARS, and RF, were employed for feature wavelength extraction. UVE, by introducing random noise variables as a reference (Kabir et al., 2023), successfully identified and eliminated uninformative wavelengths, achieving 100% accuracy in fused models such as 2nd-UVE-PLS-DA. RF, through variable importance assessment, consistently selected meaningful features (Li et al, 2025b) and exhibited stable performance across different datasets. By contrast, although CARS adaptively selects features based on the principle of survival of the fittest, its performance was highly sensitive to preprocessing (Li et al., 2009). In MIR spectra, its accuracy dropped sharply (e.g., SNV-CARS-PLS-DA declined to 45.45%), suggesting that its aggressive elimination mechanism may discard informative variables, leading to underfitting. These results emphasize that the suitability of feature selection methods depends on both the data type and the preprocessing strategy applied.

### 5.3 Synergistic advantages of spectral fusion

Spectral fusion strategies further improved classification performance. Under different preprocessing and feature selection conditions, fused models consistently outperformed single-spectrum models, confirming the strong complementarity of NIR and MIR data. NIR spectra primarily capture overtone and combination vibrations of functional groups such as O–H, C–H, and N–H, providing information on physical properties and overall chemical composition (Da et al., 2017). MIR spectra correspond to fundamental vibrations of molecular functional groups, offering detailed structural information (Liu et al., 2024). The integration of these complementary spectral domains expands the feature space and reduces the limitations inherent to single-spectral models caused by missing information or noise interference. In addition to complementarity, spectral fusion provides further advantages in terms of model robustness, prediction stability, and resistance to interference. Since NIR and MIR capture different types of chemical and physical signals, their combination reduces the model’s sensitivity to variations in sample morphology, environmental fluctuations, or preprocessing choices. For example, preprocessing methods that negatively affect one spectrum (such as SG smoothing in the NIR domain) do not compromise fused models to the same extent, because the discriminative features are distributed across multiple spectral regions. Moreover, fusion helps mitigate the impact of sample heterogeneity by allowing one spectral domain to compensate when the other is affected by local disturbances or noise. This substantially enhances the reliability, consistency, and practical applicability of the classification models. Consequently, spectral fusion significantly enhances the stability and accuracy of discriminant models, offering a more comprehensive and reliable approach to origin identification of complex traditional Chinese medicinal systems.

### 5.4 Application implications and future prospects

This study established classification models for discriminating the geographical origin of *Salvia miltiorrhiza* by integrating near infrared and mid infrared spectral data coupled with variable selection strategies. Evaluation via LOOCV demonstrated that the developed models effectively distinguished samples from the four major producing regions, confirming the feasibility and efficacy of integrating spectral fusion and chemometric approaches for tracing the origin of traditional Chinese medicinal materials.

From a methodological perspective, this work systematically examined the synergistic roles of spectral preprocessing, variable selection, and data fusion. Results revealed that suitable preprocessing methods enhanced spectral quality, variable selection identified feature wavelengths closely related to geographical origin, and the combination of NIR and MIR spectra leveraged their complementary information covering both functional group vibrations and structural fingerprint regions, thereby significantly improving model classification performance and robustness. This integrated methodology not only offers a reliable technical framework for authenticating the origin of *Salvia miltiorrhiza*, but also serves as a referential strategy for the rapid and nondestructive discrimination of other herbal medicines. It should be noted, however, that the current model validation relies primarily on internal cross-validation, and the samples were limited to four major producing regions, excluding those from minor producing areas. External validation using independent batches has not yet been conducted. Therefore, future work should emphasize the collection of new batches from the same regions as well as samples from other regions, particularly minor producing areas, to perform external validation and comprehensively assess the model’s practical applicability and generalizability.

## Data Availability

The original contributions presented in the study are included in the article/Supplementary Material, further inquiries can be directed to the corresponding authors.
