# Supplementary material for: Multisource spectral fusion combined with variable selection for rapid geographical origin discrimination of Salvia miltiorrhiza
Source: Front Chem. 2025 Dec 9;13:1730996. doi: 10.3389/fchem.2025.1730996 (PMC12722808; doi:10.3389/fchem.2025.1730996)
Supplement: Supplementary file 1 [file DataSheet1.docx]

Supplementary Material

# Supplementary Figures

**Supplementary Figure 1.** PCA score plots of near-infrared spectra established using six different spectral preprocessing methods: (a) MSC; (b) SNV; (c) MC; (d) SG; (e) 1st derivative; (f) 2nd derivative.

**Supplementary Figure 2.** PCA score plots of mid-infrared spectra established using six different spectral preprocessing methods: (a) MSC; (b) SNV; (c) MC; (d) SG; (e) 1st derivative; (f) 2nd derivative.
